# T-ISAAC: a novel TCR cloning system derived from T cells’ cis activation

**DOI:** 10.1038/s41392-022-01086-9

**Published:** 2022-07-06

**Authors:** Zehua Tian

**Affiliations:** neoX Biotech Inc., No. 4560 Jinke Road, Pudong District, Shanghai, 201210 China

**Keywords:** Adaptive immunity, Immunological techniques

In a recent article published in *Nature Biomedical Engineering*, Kobayashi and colleagues revealed a novel T cell activation induced by *cis* interaction, and developed the T-cell immunospot array assay on a chip (T-ISAAC) system that could detect antigen-specific T cells and clone the T-cell receptors (TCRs) efficiently based on this finding.^1^

The conventional T cell activation is triggered by the interaction of the TCRs on T cells and the peptide-major histocompatibility complex (pMHC) molecules on antigen presenting cells, which is called *trans* interaction.^2^ However, despite very few literatures exposing the finding of cis interaction of TCRs and pMHC molecules, this paper by Kobayashi and his colleagues clearly demonstrated a possibility of cis interaction-induced T cell activation, revealed by the auto-activated single T cell on a chip provided with endogenous or extraneous peptides. This finding is of high relevance regarding to the roles of the immune responses in human, including anti-infections and anti-cancer functions, as well as the acceleration of allergy, autoimmunity and transplant rejections.^3^

The initial work started with the detection and confirmation of *cis* interaction-induced mouse T cell activation on a microwell-array chip applying single T cell per well. The single T cell activation was confirmed with cytokines secretion for as early as 2hs post stimulation. Subsequently, the antigen-dependency of the *cis* activation of T cells was confirmed, and the binding affinity between the TCR and the pMHC complexes do influence the *cis* activation levels of T cells, indicated by assays using wild type peptides or the mutated peptides with a weaker affinity. Considering the important roles that T cells play in the immune system,^4^ it is critical to clarify two points, firstly whether the costimulatory molecules and adhesion molecules was involved in T cells’ *cis* activation; secondly whether T cells’ *cis*-activation can be induced by endogenous antigens, which imply for a cross presentation. According to Kobayashi’s data, answers to both questions are “Yes”. In line with the findings on mouse CD8 T cells, cis interactions on human CD8 T cells were confirmed, so was the engagement of costimulatory molecules and adhesion molecules. Subsequently, the cytotoxicity of the *cis*-activated human T cells was confirmed by direct killing assessment.

The confirmed *cis* interactions on T cells lead to the development of the T-ISAAC system for TCR cloning by Kobayashi et al. This four-stepped system including the detection of antigen specific T cells, retrieval of each detected cell, identification of each cell’s TCR-cDNAs and repertoire, and assessment of the identified TCRs’ function. The sensitivity of T-ISAAC has been validated and the efficacy for TCR cloning is assessed. Though with a limitation in the number of antigen models and T cell donors tested in this paper, the established models herein showed satisfying efficacy of the T-ISAAC system for antigen specific TCR screening, and primary T cells engineered with the cloned TCRs were confirmed for superior cytotoxicity functions compared to positive controls, herein TCRs already used in clinical trials.

Compared with classical strategies for antigen-specific TCR cloning, T-ISAAC stands out with its rapidness of TCR cloning and the pMHC tetramer-free TCR identification pattern. The conventional bulk cell culture and TCR cloning usually take months and have a high frequency of false positives. The pMHC tetramer-based TCR screening and cloning systems highly depend on pMHC tetramers that are with a limited commercial availability, and the in-house preparation is expensive and laborious, with unstable qualities. The combination of next-generation sequencing and single cell sequencing seems to provide comprehensive analysis to the TCR repertoire of target T cells, with advantages of high throughput process and rapidness, however, the expenses are marvelous, and costly equipments and proficient professionals are required.

However, there are some possible limiting factors for TCR cloning using this T-ISAAS system. There is a possibility that self antigen-occupied MHC excludes exogenous antigen-loading, due to the lack of knowledge in antigen selectivity of MHC molecules on T cells involved in the *cis* activation. Meanwhile, the clinical application of T-ISAAC would be impacted by the long-term in vivo antitumor activities of the generated T cells, and Kobayashi’s data had an indication for cell exhaustion of T cells generated by T-ISAAC, which is a common concern in T cell therapeutics, and multiple methods including decreasing the pre-culture period, optimizing the cell culture medium, gene engineering by knocking down or knocking out the exhaustion factors, are under investigation. Besides, given the antigen models used limited to CD8 T cells in the original paper, and the structural differences between MHCI, with only α chain anchored on the cell membranes and MHCII, with both α and β chains anchored on the cell membranes, the chances for *cis* interaction of TCR and MHCII is supposed to be lower, though the capturing of exogenous antigens of MHCII should not be hassled. Since the tonic signaling is mainly observed in T cells in lymphoid organs, and is not detectable in T cells isolated from blood, the effects of T cells’ tonic signaling in *cis* activation is not discussed.

**Fig. 1:**
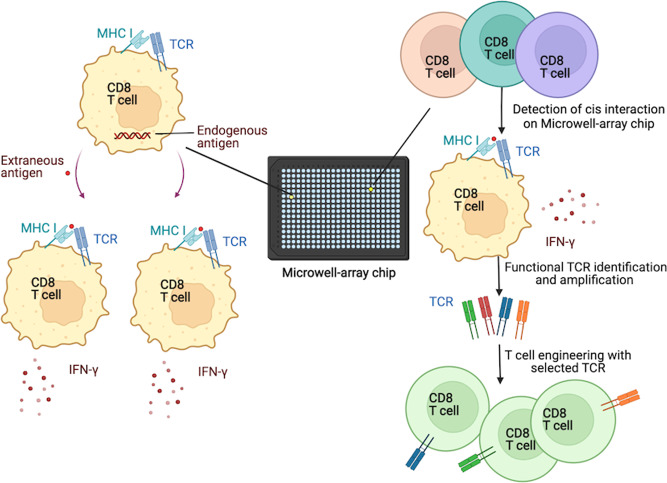
Illustration of T-ISAAC, a novel TCR cloning system derived from T cells’ *cis* activation. T cells could be activated by the *cis* interaction of TCR and MHC molecules, loaded with endogenous or extraneous antigens. Based on this finding, the T-ISAAC system that can efficiently detect and clone human antigen-specific TCRs was developed applying a microwell-array chip coated with biotinylated-cytokine-specific antibodies. The activated T cells selected by IFN-γ secretion was then retrieved and used for TCRs cloning. Then the cloned TCRs could be introduced into T cells for functional assessment. This figure was created with BioRender.com

TCR is the only molecule that senses antigen on human T cells, therefore, the investigation of the biochemical and biophysical mechanisms of the interaction of TCR and pMHC molecules is crucial.^5^ The research by Kobayashi et al had revealed the *cis* activation of T cells with a similar molecular mechanism and cytotoxic ability compared to the *trans* activation, indicating for the potential anti-tumor and anti-infections capabilities of these *cis*-activated T cells. Alongside, a rapid TCR cloning system herein nominated T-ISAAC was developed and confirmed with high efficacy in antigen-targeting TCR screening (Fig. 1). The relevant results allow for a better understanding to the complexity of the immune regulation and immune performance, as well as providing references to the smarter design for the immune therapeutic products that may target cancer, inflammation, autoimmune diseases.
